# eCPR bei postpartaler Lungenembolie

**DOI:** 10.1007/s00101-025-01553-2

**Published:** 2025-06-29

**Authors:** Marius Graf, Catalina Agripina Vaduva, Matthias Schröder, Philipp M. Lepper, Tobias Fink, Thomas Volk, Sven Oliver Schneider

**Affiliations:** 1 Klinik für Anästhesiologie, Intensivmedizin und Schmerzmedizin, Universitätsklinikum des Saarlandes, Kirrberger Straße 100, 66424 Homburg, Deutschland; 2 Zentrale Notaufnahme, Universitätsklinikum des Saarlandes, Homburg, Deutschland

## Kasuistik

Eine 36-jährige Patientin (Gravida VI, Para V) stellte sich zur elektiven Sectio caesarea in der 37 + 1 Schwangerschaftswoche vor. Anamnestisch bestanden ein insulinpflichtiger Gestationsdiabetes sowie eine familiäre Thrombophilie. Eigenanamnestisch war bislang keine Thrombose aufgetreten. Aufgrund des Body-Mass-Index von 42 kg/m^2^, des Gestationsdiabetes und der positiven Familienanamnese für Thrombophilie wurde die Schwangerschaft als Risikoschwangerschaft eingestuft. Der Kaiserschnitt erfolgte in Spinalanästhesie und verlief komplikationslos.

Am Folgetag der Sectio gab die Patientin im Rahmen der Mobilisation auf der Entbindungsstation plötzliche massive Dyspnoe an und synkopierte. Die Patientin wurde auf die gynäkologische Überwachungsstation verlegt. Sie war tachykard (bis 160 bpm), hypoton (50/30 mm Hg), kaltschweißig und hypoxisch.

Kurz darauf verlor die Patientin das Bewusstsein, und es war kein Puls mehr tastbar – unverzüglich wurden Reanimationsmaßnahmen begonnen. Bei Eintreffen des klinikinternen Notfallteams wurde eine mechanische Reanimationshilfe etabliert. Eine orientierende Echokardiographie zeigte ein deutlich dilatiertes rechtes Herz. Ein NIRS-Monitoring wurde während der Reanimation nicht durchgeführt. In Zusammenschau mit einer weiterhin bestehenden PEA wurde die Verdachtsdiagnose der LAE gestellt. Das Team entschied sich zur Durchführung einer Thrombolyse mit Alteplase und alarmierte das ECPR-Team, das 53 min nach dem Reanimationsbeginn eine va-ECMO etablierte. Bei ausbleibendem ROSC wurde eine CT-Diagnostik durchgeführt; diese bestätigte die Diagnose einer LAE. Ein hämodynamisch relevanter Perikarderguss wurde unverzüglich drainiert und die Patientin auf eine Intensivstation verlegt.

Bei dort steigendem Vasopressorenbedarf trotz va-ECMO und sonographisch dargestellter freier abdomineller Flüssigkeit wurde die Patientin kurz darauf im hämorrhagischen Schock laparotomiert. Als Blutungsquelle fand sich ein blutender Uterus, sodass man sich zur Hysterektomie entschloss. Bei weiterhin bestehender Kreislaufinstabilität fand sich intraabdominell zusätzlich eine Leberlazeration entlang des Lig. falciforme, die operativ versorgt wurde. Nach Transfusion von 13 EK, 15 FFP, 7 TK, 2000 Einheiten PPSB, 2500 IE Faktor XIII sowie Fibrinogen (10 g) konnte die Patientin mit stabilisierten Kreislaufverhältnissen wieder auf die ICU verlegt werden. Im Verlauf der Behandlung zeigten sich am Folgetag beidseits lichtstarre Pupillen. Im cCT wurden ein generalisiertes Hirnödem und eine beginnende untere Einklemmung festgestellt. Bei infauster Prognose wurde mit den Angehörigen der Patientin eine Therapiezieländerung besprochen, und die Patientin verstarb am dritten postoperativen Tag.

## Diskussion

Verglichen mit einer vaginalen Geburt weist die Sectio eine höhere Inzidenz an postpartalen Lungenembolien auf (Kaiserschnitt: 5,1/1000 Geburten vs. vaginale Geburt 1,57/1000 Geburten) [8].

Die LAE ist damit eine seltene (> 1 ‰) peri- und postpartale Komplikation [10], stellt jedoch die häufigste mütterliche Todesursache in Industrieländern dar (10–15 %) [14]. Pathophysiologisch zählt sie zur obstruktiven Schockform. Ein Thrombus verlegt hierbei die pulmonalarterielle Strombahn, und es kommt bei größerem Befund zu einer rechtsventrikulären Druckbelastung.

Bei hämodynamisch instabilen Patienten mit Verdacht auf eine Lungenembolie stellt die bettseitige Sonographie – insbesondere die transthorakale Echokardiographie (TTE) sowie die Kompressionssonographie der Beinvenen – eine entscheidende diagnostische Maßnahme dar [9]. Als besonders spezifisches Echokardiographie-Zeichen gilt die rechtsventrikuläre Dilatation, welche im klinischen Kontext ein zentrales Kriterium zur Diagnosestellung darstellen kann [9].

Die therapeutische Konsequenz bei instabilen Patienten besteht in der schnellstmöglichen Durchführung einer Reperfusionstherapie. Der Goldstandard ist dabei die systemische Thrombolyse (Empfehlungsgrad I [9]), sofern keine Kontraindikationen bestehen. Absolute Kontraindikationen umfassen beispielsweise größere operative Eingriffe innerhalb der letzten 3 Wochen, während die erste postpartale Woche als relative Kontraindikation gilt [9].

Bei persistierender hämodynamischer Instabilität oder kardiopulmonalem Stillstand kann die Etablierung einer venoarteriellen extrakorporalen Membranoxygenierung (va-ECMO) im Sinne eines „bridging to reperfusion“ erfolgen. Hierbei dient die ECMO als Überbrückung bis zur Durchführung einer chirurgischen oder kathetergestützten Thrombektomie. Neuere Studien berichten von vergleichbaren Mortalitätsraten zwischen chirurgischer (28 %) und kathetergestützter Intervention (25 %), wobei Letztere bislang auf einer noch begrenzten Datenlage beruhen [1]. Im Sinne eines ‚Bridge-to-Intervention‘-Ansatzes kann der frühzeitige Einsatz einer ECMO bei fulminanter Lungenarterienembolie eine therapeutische Option darstellen. Ein potenzieller Vorteil dieses Vorgehens besteht in der Möglichkeit, auf eine systemische Thrombolyse zu verzichten und somit deren assoziierte Komplikationen zu vermeiden [1, 17].

Im Rahmen einer Reanimation ist die Organperfusion durch Thoraxkompressionen allein eingeschränkt, da das hierdurch erzielte Herzzeitvolumen lediglich 25–30 % des physiologischen Ausgangswerts beträgt [4]. Durch Implementierung einer extrakorporalen kardiopulmonalen Reanimation (eCPR) mittels ECMO kann die Perfusion zentraler Organe signifikant verbessert werden, was zu einer potenziellen Verbesserung von Überleben und neurologischem Outcome führt [12]. Die Anwendung der eCPR ist insbesondere bei reversiblen kardialen Ursachen indiziert (Empfehlungsgrad IIb) und sollte idealerweise innerhalb von 60 min nach Eintritt des Kreislaufstillstands initiiert werden, um einen prognostischen Nutzen zu erzielen [16].

Nach der Thrombolyse wird eine verlängerte Reanimation empfohlen [12], weshalb die Hinzunahme einer mechanischen Reanimationshilfe sinnvoll sein kann. Weltweit ist eine Nutzungszunahme der mechanischen Reanimationshilfen (mCPR) zu beobachten [7]. MCPR führen häufiger zu Verletzungen als manuell durchgeführte Reanimationsmaßnahmen (Risikozunahme: ca. 36 %) [16]. Am häufigsten treten Rippenfrakturen oder andere skeletale Verletzungen des Thorax auf (Prävalenz ca. 60 %) [19]. Auch kardiale Verletzungen im Rahmen einer kardiopulmonalen Reanimation sind nicht selten. Je nach Studienlage finden sich bei bis zu 50 % der obduzierten Patienten Hinweise auf strukturelle Herzverletzungen [5]. Die genaue Pathogenese des in der vorliegenden Kasuistik beschriebenen hämodynamisch relevanten Perikardergusses lässt sich retrospektiv nicht abschließend klären; es erscheint jedoch plausibel, dass dieser im Sinne einer Contusio cordis im Kontext der mechanischen Reanimationsmaßnahmen entstanden sein könnte.

Leberverletzungen, wie in der Kasuistik beschrieben, treten in ungefähr 2–5 % der Fälle nach einer mCPR auf. Sehr häufig entstehen dabei Leberhämatome [6, 15]. Es ist zu vermuten, dass aufgrund des auch noch postpartal vergrößerten Uterus die Leber nach kranial verschoben war. Eine Verletzung der Leber durch eine mCPR bei der Reanimation einer Schwangeren ist daher wahrscheinlicher. Entscheidet man sich für eine mCPR, sollten die mitgelieferten Befestigungsmaterialen, trotz ggf. entstehendem kurzem Zeitnachteil, genutzt werden, um die Einhaltung des korrekten Druckpunkts, auch bei Lageveränderungen der Patienten, zu garantieren.

Das neurologische Outcome nach einer Reanimation hängt wesentlich von der zerebralen Oxygenierung und Perfusion ab. In der Kardiochirurgie wird hierfür häufig die Near-Infrared Spectroscopy (NIRS) eingesetzt, um die regionale Sauerstoffsättigung (rSO_2_) frontal am Stirnlappen zu messen. Studien zeigen, dass höhere rSO_2_-Werte während der CPR mit einem ROSC und einem besseren neurologischen Outcome assoziiert sind [2, 11, 18]. In den aktuellen Leitlinien von AHA und ERC ist NIRS jedoch noch nicht zur Reanimation empfohlen, weshalb diese im vorliegenden Fallbeispiel auch nicht durchgeführt wurde.

Nach einer Thrombolyse scheint das Überleben der postpartalen Patientinnen grundsätzlich gut zu sein (84 %), allerdings entwickeln ca. 60 % der Patientinnen schwere vaginale oder intraabdominelle Blutungen [9], so auch die obige Patientin. Die Therapie eines solch massiven Blutverlustes gelingt oft nur mittels Massivtransfusion, welche durch eine Point-of-Care(POC)-Gerinnungstherapie optimiert werden sollte. Die Nutzung von POC-Verfahren wird leitlinienübergreifend für die Massenblutung empfohlen (Empfehlungsgrad 1C) [13]. Im vorliegenden Fall galt es, den Mittelweg zu finden zwischen Gerinnungsoptimierung bei akuter Blutung und einer notwendigen Antikoagulation nach der LAE. Innerhalb der ersten 24 h nach einer ECMO-Anlage kann noch auf eine Antikoagulation verzichtet werden [3].

Trotz der sofort begonnenen Reanimation, Lyse und Implantation der eCPR sowie der darauffolgenden Massivtransfusion ist die Patientin letztlich verstorben. Im vorliegenden Fall wies die Patientin trotz praktisch minimaler No-Flow Time einen schweren hypoxischen Hirnschaden auf. Dieser ist vermutlich durch die initial massive Thrombusverlegung mit konsekutiver Hypoxämie trotz Thoraxkompressionen und Kreislaufinstabilität bei Massenblutung zu erklären.

## Fazit für die Praxis

- Die postpartale Lungenembolie ist eine seltene, aber häufig tödliche Komplikation. Das Risiko ist innerhalb der ersten Tage postpartum am höchsten.
- Die Durchführung einer Lyse nach einer Sectio ist die Entscheidung gegen eine absolute Kontraindikation. Alternative Verfahren wie die chirurgische oder interventionelle Reperfusion sind immer in Betracht zu ziehen.
- Bei einer Reanimation mit vermuteter ursächlicher LAE soll eine Thrombolyse gegen die Möglichkeit einer chirurgischen oder kathetergestützten Thrombektomie abgewogen werden. Der Einsatz einer ECMO kann im Sinne einer „bridge to intervention“ erwogen werden.
- Bei der mCPR sollte unbedingt auf einen korrekten Druckpunkt geachtet werden.
- Trotz optimaler Versorgung ist ein schicksalhafter Verlauf immer möglich.

## Abkürzungen

ECMO: Extrakorporale Membranoxygenierung
eCPR: Extrakorporale kardiopulmonale Reanimation
LAE: Lungenarterienembolie
mCPR: Mechanische kardiopulmonale Reanimation im Sinne einer mechanischen Kompressionshilfe
NIRSMonitoring: Near-Infrared-Spectroscopy-Monitoring
POC: Point-of-Care
TTE: Transthorakale Echokardiographie
